# Geometrical Consistency Modeling on B-Spline Parameter Domain for 3D Face Reconstruction From Limited Number of Wild Images

**DOI:** 10.3389/fnbot.2021.652562

**Published:** 2021-04-13

**Authors:** Weilong Peng, Yong Su, Keke Tang, Chao Xu, Zhiyong Feng, Meie Fang

**Affiliations:** ^1^School of Computer Science and Cyber Engineering, Guangzhou University, Guangzhou, China; ^2^Tianjin Key Laboratory of Wireless Mobile Communications and Power Transmission, Tianjin Normal University, Tianjin, China; ^3^Cyberspace Institute of Advanced Technology, Guangzhou University, Guangzhou, China; ^4^College of Intelligence and Computing, Tianjin University, Tianjin, China

**Keywords:** 3D face modeling, B-spline, face reconstruction, geometrical consistency, parametric domain

## Abstract

A number of methods have been proposed for face reconstruction from single/multiple image(s). However, it is still a challenge to do reconstruction for limited number of wild images, in which there exists complex different imaging conditions, various face appearance, and limited number of high-quality images. And most current mesh model based methods cannot generate high-quality face model because of the local mapping deviation in geometric optics and distortion error brought by discrete differential operation. In this paper, accurate geometrical consistency modeling on B-spline parameter domain is proposed to reconstruct high-quality face surface from the various images. The modeling is completely consistent with the law of geometric optics, and B-spline reduces the distortion during surface deformation. In our method, 0th- and 1st-order consistency of stereo are formulated based on low-rank texture structures and local normals, respectively, to approach the pinpoint geometric modeling for face reconstruction. A practical solution combining the two consistency as well as an iterative algorithm is proposed to optimize high-detailed B-spline face effectively. Extensive empirical evaluations on synthetic data and unconstrained data are conducted, and the experimental results demonstrate the effectiveness of our method on challenging scenario, e.g., limited number of images with different head poses, illuminations, and expressions.

## 1. Introduction

**Figure 1:**
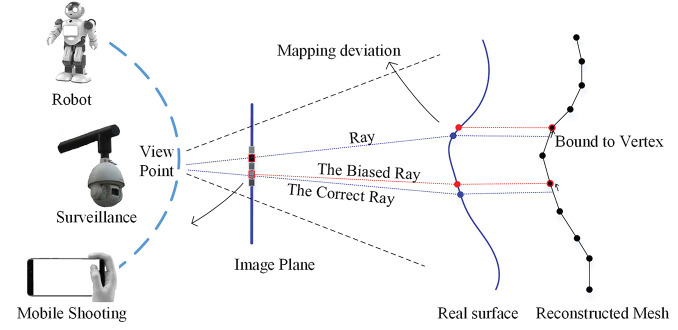
Geometric optics of BP (i.e., back projection) imaging on two types of surfaces: the correct ray lines go through the blue points on the true shape, while the biased ones go through red points on the mesh shape because the cross point between a ray and mesh is bounded to vertex. The difference between red point and the blue point is referred to local mapping deviation.

3D face has been extensively applied in the areas of face recognition (Artificial and Aryananda, 2002; Mian et al., 2006), expression recognition (Zhang et al., 2015). These face analysis technologies are of significance for human-robot cooperative tasks in a safe and intelligent state (Maejima et al., 2012). So 3D face reconstruction is a import topic, and it is meaningful to reconstruct specific 3D face from person-of-interest images under many challenge scenes. The images under challenge scene are also referred as images in the wild, having following characteristics: (1) significant changes in illuminations across time periods; (2) various face poses caused by different camera sensors and view points; (3) different appearances among different environment; (4) occlusions or redundant backgrounds. More seriously, only limited number of identity images are available under human-robot interaction, surveillance, and mobile shooting scenario as listed in Figure 1, sometimes.

As a whole, reconstruction technologies include single-image method, multiple images, and even unconstrained images based methods. Recent researches (Kemelmacher and Seitz, 2011; Roth et al., 2015, 2016) prove that good reconstruction depends on two aspects of efforts: (1) enough rich local information, e.g., normal, and (2) a good face prior, e.g., face template. Particularly, the latter is to find an embedding representation with good characteristic to register local information finely.

According to the template representation, these methods can be categorized into three classes: (i) methods without using template, e.g., integration (Kemelmacher and Seitz, 2011) and structure from motion (Koo and Lam, 2008), (ii) methods using a single discrete template, e.g., a reference face mesh (Roth et al., 2015), and (iii) methods using a statistic continuous template, e.g, T-splineMMs (Peng et al., 2017), or discrete template, e.g., 3DMMs (Piotraschke and Blanz, 2016; Roth et al., 2016). The methods with template always generate good global shape compared with those without template, and a statistic template contributes to a better personalization. Therefore, it is very significant to find a excellent template representation for face reconstruction. Mesh model is widely used due to its rapid computation and popularity in computer vision, but it is not well-compatible with geometric optics in vertex level, resulting in local mapping deviation of rays, seen in Figure 1. This makes local information not strictly registered physically. Additional, discretization of Laplace-Beltrami operation (LBO), i.e., cotangent scheme (Meyer et al., 2003), may bring a deformation distortion at local, which often happens when images are not enough for high-quality normal estimation. This distortion irregularly occurs at the edge and the location with large curvature changing, e.g., nose and mouth. Lastly the topology-fixed mesh also restricts an extended refinement. All above problem limits reconstruction precision of mesh.

To solve the existing issue in mesh template, we adopt classic B-spline embedding function (Piegl and Tiller, 1997) to register local information and reconstruct face. Firstly, B-spline surface is a parametric surface that can approximate the true shape of an object with fewer parameters (control points) than mesh. It contributes to correct rays in geometric optics, that makes local information, i.e., texture, feature points and normals, accurately registered. Secondly, we use 2nd-order partial derivative operator w.r.t. parameters as the local deformation constraint to reduce the deformation distortion. Lastly, B-spline surface also can be used to generate mesh in any precision or be extended for further refinement. The three characteristics of B-spline face show great advantages over a mesh template based method. Given a collection of images, we use B-spline embedding function as 3D face representation and model 0th- and 1st-order consistency of reconstruction in the parameter domain, which makes BP imaging rays completely compatible with geometric optics. The 0th-order consistency model guarantees that the images are well-registered to surface even if the face images has occlusion or expression; And the 1st-order consistency model guarantees that the surface normals is consistent to the normals estimated from images. Both qualitative and quantitative experiments are conducted and compared with other methods.

In a nutshell, there are two primary contributions:

1. Pinpoint geometrical consistency is modeled on B-spline embedding function for face reconstruction from multiple images, completely consistent with the law of geometric optics.
2. 0th- and 1st-order consistency conditions and its a practical solution is proposed to optimize B-spline face effectively, which is able to handle variations such as different poses, illuminations, and expressions with limited of number images.

In the following, we will first review related work in section 2. Section 3 provides a geometric modeling of multiple BP imaging in image-based stereo for our problem. We introduce the B-spline embedding and its brief representations in section 4 and present consistency modeling for B-spline face reconstruction in section 5. In addition, a practical solution is proposed in section 6. We conduct experiment in section 7 and conclude in section 9.

## 2. Related Work

### 2.1. 3D Face Required Scenes

With the development of robots and AIoT (Qiu et al., 2020), vision will play an very important role in safety (Khraisat et al., 2019; Li et al., 2019), scene and human understanding (Zhang et al., 2015; Meng et al., 2020). As a base technology, 3D face contributes to the scenes greatly. For example, to build humanoid robots that interact in a human-understanding manner, automatic face, and expression recognition is very import (Zhang et al., 2015). The recognition during real-life human robot interaction could still be challenging as a result of subject variations, illumination changes, various pose, background clutter, and occlusions (Mian et al., 2006). However, humanoid robot API of original version cannot always be able to handling such challenges. Optimal, robust, and accurate automatic face analysis is thus meaningful for the real-life applications since the performance of facial action and emotion recognition relies heavily on it. Many parametric approaches like 3DMMs (Blanz and Vetter, 1999; Blanz et al., 2004) and face alignment with 3D solution (Zhu et al., 2016) in the computer vision field have been proposed to estimate head pose, recognition identity, and expression from real-life images to benefit subsequent automatic facial behavior perception to address the above issues. Therefore, 3d face modeling in a humanoid robot view is of great significant to handling the challenging face analysis during interaction.

### 2.2. 2D Images Based Face Reconstruction

2D methods generally cover several kinds of fundamental methods including Structure from Motion (SFM) (Tomasi and Kanade, 1992), Shape from Shading (SFM) (Zhang et al., 1999), 3D Morphable Model (3DMM) (Blanz and Vetter, 1999; Blanz et al., 2004), and Deep learnings (Richardson et al., 2017; Deng et al., 2019). SFM methods compute the positions of surface points based on an assumption that there exists a coordinate transformation between the image coordinate system and the camera coordinate system. And SFS methods compute surface normals with an assumption that the subject surface is of Lambertian and under a relatively distant illumination. And the idea of 3DMM is that human faces are within a linear subspace, and that any novel face shape can be represented by a linear combination of shape eigenvectors deduced by PCA. SFS and SFM give the geometrical and physical descriptions of face shape and imaging, and 3DMM concentrates on the statistical explanation of 3D meshes or skeletons. Deep learning methods infer 3D face shape or texture (Lin et al., 2020) by statistically learning mapping between face images and their 3D shapes (Zhou et al., 2019). Being limited to data size, most of them relies 3DMM or PCA for synthesizing supplementary ground truths (Richardson et al., 2016) or as a priori (Tran et al., 2017; Gecer et al., 2019; Wu et al., 2019), resulting absence of shape detail. It's believed that face reconstruction is rather a geometrical optimization problem than a statistical problem, as 3DMM is more suitable to be an assistant of the geometrical method when building detailed shape, e.g., that by Yang et al. (2014).

### 2.3. Shape in Shading and Structure in Motion

SFS has been widely used for reconstruction, e.g., single-view reconstruction (Kemelmacher Shlizerman and Basri, 2011), multiple frontal images based reconstruction (Wang et al., 2003), and unconstrained image based reconstruction (Kemelmacher and Seitz, 2011; Roth et al., 2015). As single-view is ill posed (Prados and Faugeras, 2005), a reference is always needed (Kemelmacher Shlizerman and Basri, 2011). For unconstrained images, photometric stereo is applied to obtain accurate normals locally (Kemelmacher and Seitz, 2011; Roth et al., 2015). SFM uses multiple frame or images to recover sparse 3D structure of feature points of an object (Tomasi and Kanade, 1992). Spatial-transformation approach (Sun et al., 2013) only estimates the depth of facial points. Bundle adjustment (Agarwal et al., 2011) fits the large scale rigid object reconstruction, but it cannot generate the dense model of non-rigid face. Incremental SFM (Gonzalez-Mora et al., 2010) is proposed to build a generic 3D face model for non-rigid face. The work by Roth et al. (2015) optimizes the local information with normals from shading, based on a 3D feature points-driven global warping. Therefore, shading and motion are important and very distinct geometric information of face, and they enhance the reconstruction when being combined. In our method, 0th- and 1st-order consistency of stereo is modeled to integrate the advantages of both shading and motion information.

### 2.4. Facial Surface Modeling

Surface modeling is dependent on the data input (point cloud, noise, outlier, etc), output (point cloud, mesh, skeleton), and types of shape (man-made shape, organic shape). Point cloud, skeleton, and mesh grid are the widely used man-made shape type for face reconstruction. Lu et al. (2016) present an a stepwise tracking method approach to reconstruct 3D B-spline space curves from planar orthogonal views through minimizing the energy function with weight values. Spatial transformation method (Sun et al., 2013) estimates positions of sparse facial feature points. Bundle adjustment builds the dense point cloud for large scale rigid object with a great number of images (Agarwal et al., 2011). Heo and Savvides (2009) reconstruct face dense mesh based on skeleton and 3DMM. Kemelmacher and Seitz (2011) apply integration of normals to get discrete surface points, which may produce incredible depth when the recovered normals are unreliable. Roth et al. (2015) reconstruct face mesh based on Laplace mesh editing, which may produce local mesh distortion after several iterations of local optimization. In work of mesh reconstruction, surface-smoothness priors is also needed to guarantee the smoothness of discrete mesh based on point cloud, e.g., radial basis function (Carr et al., 2001) and Poisson surface reconstruction (Kazhdan et al., 2006). Due to the fact that the point cloud and 3D mesh are discontinuous geometric shape, they cannot approximate the true shape of a face of arbitrary precision. There have been works of fitting B-splines to noisy 3D data, like Hoch et al. (1998). B-spline face model is a continuous free-form surface that can be reconstructed from images directly, instead of intermediate point data, but it is not a detailed model by only using structure optimization (Peng et al., 2016). Because B-spline surface is a special case of NURBS (Non-Uniform Rational B-Spline) (Piegl and Tiller, 1997), it can also be imported to 3D modeling software like Rhino3D for further editing, analysis, and transformation conveniently by adjusting the B-spline control points. It can also be converted into mesh model with any precision according to appropriate parameter interval, conveniently, which is meaningful for a system with limited memory.

## 3. Geometric Modeling

**Figure 2:**
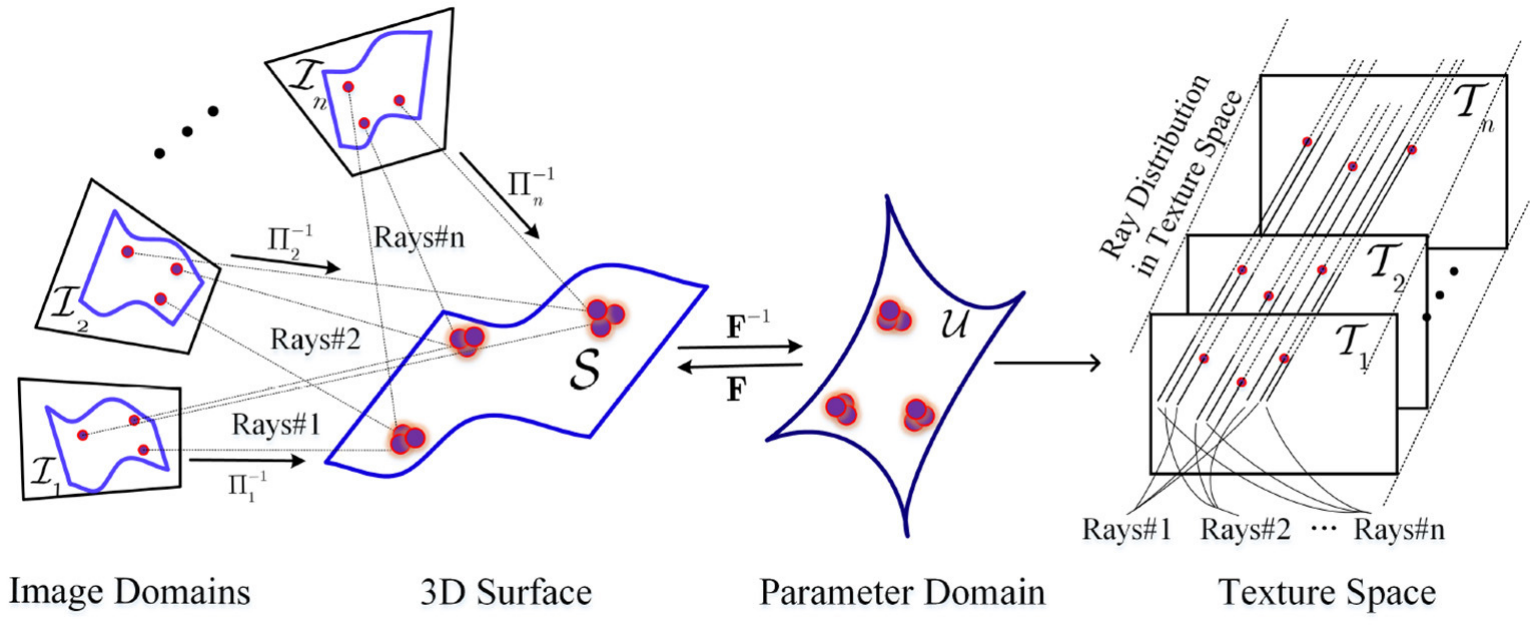
Geometric optics of multiple BP imaging.

Our problem modeling is illustrated in Figure 2. The domain of input image *I*_*i*_ from a camera is $I_{i}\subset {\mathbb{R}}^{2},i=1,2,\ldots ,n$. Π^−1^ denotes the inverse operator of Π. The camera operator $\Pi_{i}\in C^{\infty }\left({\mathbb{R}}^{3},{\mathbb{R}}^{2}\right)$ map a point P∈S to $p=\Pi_{i}\left(P\right)\in I_{i}$ using weak perspective projection, *i* = 1, 2, …, *n*. And $\Pi_{i}^{-1}$ determines the ray cluster Rays#*i* of BP imaging from $I_{i},i=1,2,\ldots ,n$. Let *s*_*i*_, *R*_*i*_, and *t*_*i*_ denote scale, rotation, and translation parameter in projection Π_*i*_. The *i*th projection operation is simply

(1) $$\begin{array}{r} \Pi_{i}\left(P\right)≜s_{i}\cdot R_{i,\left[1,2\right]}\cdot P+t_{i}. \end{array}$$

*R*_*i*, [1,2]_ expresses the first two rows of *R*_*i*_.

Let $U\subset {\mathbb{R}}^{2}$ denote the parameter domain of human face surface. A certain embedding $\text{F}\in C^{1}\left(U,{\mathbb{R}}^{3}\right)$ maps a point u∈U to the 3D point P∈S. **F**^−1^ denote the inverse operator of **F**. It is thus clear that different embedding *F* determine different face shapes. According to the geometric optics of BP imaging, a image point $p\in I_{i}$ is back projected onto a point $\text{u}=\tau_{i}\left(p\right)\in U$ *via* the operator

(2) $$\begin{array}{r} \tau_{i}≜{\text{F}}^{-1}◦\Pi_{i}^{-1}. \end{array}$$

Therefore, an image *I*_*i*_ in the *i*-th view is mapped to surface *S*, and then is mapped to texture space by

(3) $$\begin{array}{r} T_{i}≜I_{i}◦\tau_{i}^{-1}, \end{array}$$

where we define

(4) $$\begin{array}{r} \left(I◦\tau^{-1}\right)\left(\text{u}\right)≜I\left(\Pi \left(\text{F}\left(\text{u}\right)\right)\right),\;for\;\text{u}\in U. \end{array}$$

In fact, τ_*i*_, *i* = 1, 2, …, *n* generate discrete and inconsistent rays mapping in texture space because of the discrete and different images domains, as well as the noises, seen in Figure 2.

### 3.1. 0th- and 1st-Order Consistency

Generally, the problem is how to determine **F** according to from multiple images. If all images are the captures of a same S, all ${\left\{T_{i}\right\}}_{i=1:n}$ in texture space are hoped to be highly consistent in the geometry.

First, that satisfies

(5) $$\begin{array}{r} <\hat{\text{F}},\left\{{\hat{\Pi }}_{i}\right\}>=\underset{\text{F},\{\Pi_{i}\}}{\arg\min},\text{ rank}\left(\left[\text{vec}\left(T_{1}\right),\text{vec}\left(T_{2}\right),\ldots ,\text{vec}\left(T_{n}\right)\right]\right), \end{array}$$

with $T_{i}={\left(I_{i}◦\tau_{i}^{-1}\right)}^{\#},i=1,2,\ldots ,n$. And (·)^#^ is a composition operator of fitting and sampling, to handle the inconsistency. It firstly fits a texture function based on the discrete texture and parameters mapped from one image, and then samples texture intensity values at unified parameter points {**u**_*j*_}_*j*=1 : *N*_*p*__.

**Figure 3:**
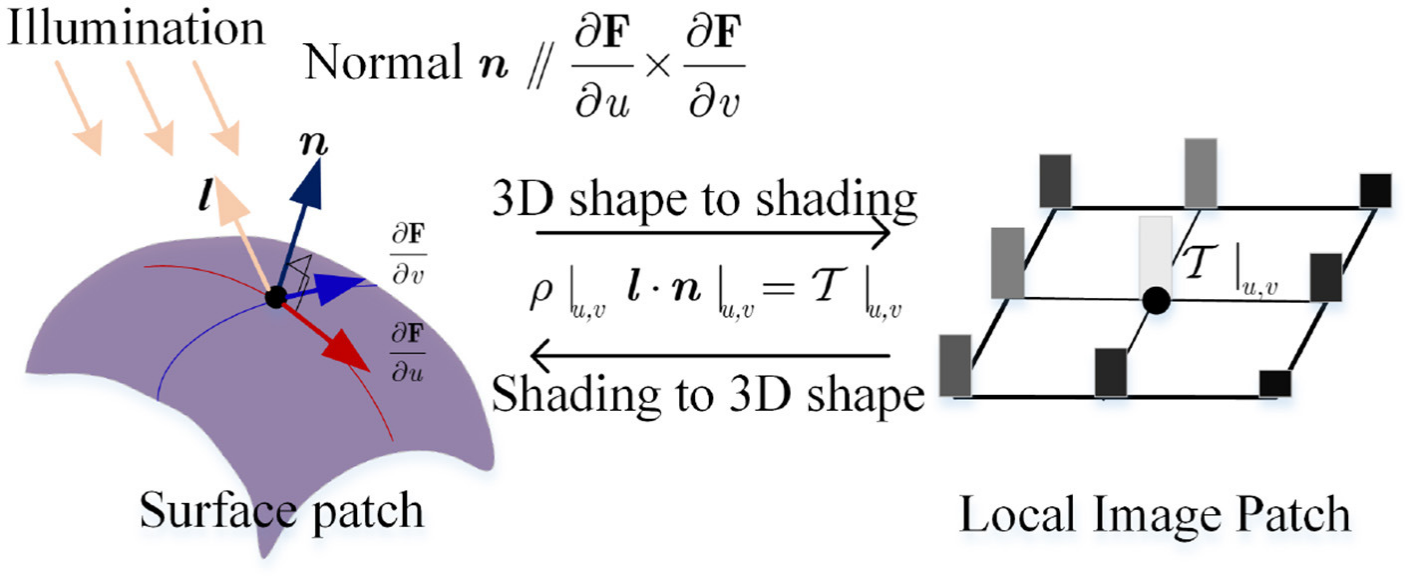
The consistency mapping equivalence between embedding **F** and the image intensity.

Second, it satisfies

(6) $$\begin{array}{r} \{\begin{array}{l} \frac{\frac{\partial \text{F}}{\partial u}\times \frac{\partial \text{F}}{\partial v}}{\left||\frac{\partial \text{F}}{\partial u}\times \frac{\partial \text{F}}{\partial v}|\right|}=n,\\ \rho_{j}n_{j}\cdot l_{i}=T_{i}{|}_{{\text{u}}_{j}}. \end{array} \end{array}$$

which describes the equivalence relation between normal ***n*** and 1st-order partial derivative in the first formulation, and the equivalence relation among albedo ρ, normal ***n***, light direction ***l***, and image intensity T in the second. This follows a linear photometric model, as seen in Figure 3.

We refer to Equations (5) and (6) as 0th- and 1st- order consistence equations in 3D surface reconstruction respectively. Generally, researchers solve any one of the two consistence problem to reconstruct 3D surface, classically, by multi-view stereo (Seitz et al., 2006) for 0th-order consistence problem, or by photometric stereo (Barsky and Petrou, 2003) for the 1st-order one.

### 3.2. Embedding **F**

There are several types of representation for embedding **F**, such as discrete mesh and *C*^2^ parametric surface. In fact the representation type of **F** also affects the reconstruction effect. Intuitively for mesh, on one hand there exists mapping deviation of rays from image points to vertices of mesh, which contributes to inaccurate texture charts ${\left\{T_{i}\right\}}_{i=1:n}$ and affects the accuracy of reconstruction. On the other, discrete differential operator, i.e., LBO (Meyer et al., 2003), brings potential distortion error when there exists obtuse triangles in the mesh caused by error local normal. Additionally, the precision of mesh also limit the detail of reconstruction.

We consider to apply *C*^2^ parametric surface as the representation of face. Generally, B-spline surface is recommended because of its advantages of good locality over other types of surfaces such as polynomial surface and Bessel surface. By B-spline surface, it doesn't exist mapping deviation in geometric optics, and it avoids the potential distortion brought by discrete differential operator. Therefore, accurate and continuous back projection texture charts ${\left\{T_{i}\right\}}_{i=1:n}$ can be generated based on Equations (2), (3), and (5). Then accurate reconstruction can be implemented based on Equation (6). What's more, the precision can be enhanced for high-detailed reconstruction by inserting control points.

## 4. B-Spline Face Embedding F, and the 0th-, 1st-, 2nd–Order Representation

The human face is assumed to be a uniform B-spline surface S of degree 4 × 4, with **B** = {***b***_*mn*_}_*M*×*N*_ as its control points. In parameter domain U, knots $U={\left\{u_{m}\right\}}_{m=1}^{M+4}$ and $V={\left\{v_{n}\right\}}_{n=1}^{N+4}$ split *uv* parameter plane into uniform grid. Let **u** denote parameter point (*u, v*). The surface function is

$$\text{F}\left(\text{u}\right)=\overset{M}{\underset{m=1}{\sum }}\overset{N}{\underset{n=1}{\sum }}R_{m,n}\left(\text{u}\right)b_{mn},$$

with *R*_*m,n*_(**u**) = *N*_*m*,4_(*u*) · *N*_*n*,4_(*v*) and

$$\{\begin{array}{c} N_{i,1}\left(w\right)=\{\begin{array}{ll} 1 & u_{i}\leq w<u_{i+1},\\ 0 & otherwise, \end{array}\\ N_{i,j}\left(w\right)=\frac{\left(w-u_{i}\right)\cdot N_{i,j-1}\left(w\right)}{u_{i+j-1}-u_{i}}+\frac{\left(u_{i+j}-w\right)\cdot N_{i+1,j-1}\left(w\right)}{u_{i+j}-u_{i+1}},\left(j=4,3,2\right). \end{array}$$

**F** is *C*^2^, meaning that it can approximate the true shape in arbitrary *uv* precision with deterministic *k*-ordered partial derivative $\frac{\partial^{k}\text{F}}{\partial u^{k}}$ and $\frac{\partial^{k}\text{F}}{\partial v^{k}}$, *k* = 1, 2, and $\frac{\partial^{2}\text{F}}{\partial u\partial v}$.

### 4.1. 0th-Order Representation

We give a more brief formulation of 0th-order representation as follows:

(7) $$\begin{array}{r} \text{F}{|}_{\text{u}}=\text{T}{|}_{\text{u}}\cdot \text{b}, \end{array}$$

where **b** denotes a 3*MN* × 1 vector storing B-spline control points, and **T**|_**u**_ denotes a sparse 3 × 3*MN* matrix stacking the 0th-order coefficients at parameter u∈U.

**Figure 4:**
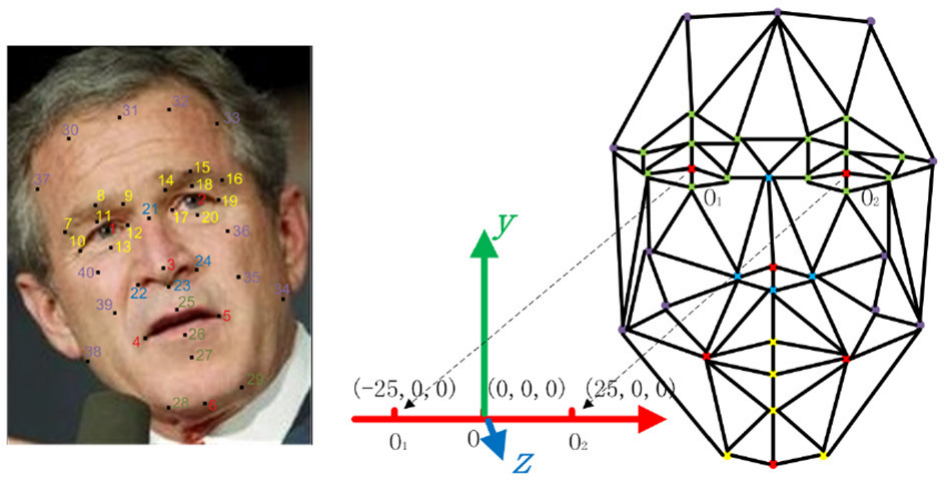
Face structure defined by 40 feature points: the left side shows the point positions in a face image; the right side shows the structure topology with eye center points of *O*_1_ (−25, 0, 0) and *O*_2_ (25, 0, 0) in 3*D* space, which looks like a frontal 2D face structure from the direction of normal (0, 0, 1). (The face image used in the figure comes from LFW database^1^).

In fact, we needn't consider all 3D points mapping to 2D images when estimating a operator Π. Instead, we only consider *f* landmark points on human face as shown in Figure 4, and their brief formulation is

(8) $$\begin{array}{r} \text{F}{|}_{\text{u}\left(l_{i}\right)}=\text{T}{|}_{\text{u}\left(l_{i}\right)}\cdot b,i=1,2,\ldots ,f, \end{array}$$

where **u**(*l*_*i*_) is the parameter point of the *i*-th feature point, *i* = 1, 2, …, *f*. The landmarks cover a sparse structure of face.

### 4.2. 1st-Order Representation

The 1st-order partial derivatives of **F** w.r.t *u* and *v* are

$$\begin{array}{l} {\text{F}}_{u}^{\prime }\left(\text{u}\right)=\overset{M}{\underset{m=1}{\sum }}\overset{N}{\underset{n=1}{\sum }}N_{m,4}^{\prime }\left(u\right)\cdot N_{n,4}\left(v\right)b_{mn}\\ =\overset{M}{\underset{m=1}{\sum }}\overset{N}{\underset{n=1}{\sum }}\left(\frac{4}{u_{m+4}-u_{i}}N_{m,3}^{\prime }\left(u\right)-\frac{4}{u_{m+5}-u_{m+1}}N_{m+1,3}^{\prime }\left(u\right)\right)\cdot N_{n,4}\left(v\right)b_{mn} \end{array}$$

and

$$\begin{array}{l} {\text{F}}_{v}^{\prime }\left(\text{u}\right)=\overset{M}{\underset{m=1}{\sum }}\overset{N}{\underset{n=1}{\sum }}N_{m,4}\left(u\right)\cdot N_{n,4}^{\prime }\left(v\right)b_{mn}\\ =\overset{M}{\underset{m=1}{\sum }}\overset{N}{\underset{n=1}{\sum }}N_{m,4}\left(u\right)\cdot \left(\frac{4}{u_{n+4}-u_{n}}N_{n,3}^{\prime }\left(v\right)-\frac{4}{u_{n+5}-u_{n+1}}N_{n+1,3}^{\prime }\left(v\right)\right)b_{mn} \end{array}$$

respectively.

Similarly, we give a more brief formulation of 1st-order partial derivative as follows:

(9) $$\begin{array}{r} \{\begin{array}{l} {\text{F}}_{u}^{\prime }{|}_{\text{u}}={\text{T}}_{1}{|}_{\text{u}}\cdot \text{b}\\ {\text{F}}_{v}^{\prime }{|}_{\text{u}}={\text{T}}_{2}{|}_{\text{u}}\cdot \text{b} \end{array}, \end{array}$$

where **T**_1_|_**u**_ and **T**_2_|_**u**_ denote the matrixes stacking the 1st-order coefficients w.r.t *u* and *v*, respectively.

Therefore, the surface normal vector at **u** can be computed by the cross product

(10) $$\begin{array}{r} n{|}_{\text{u}}=\frac{{\text{F}}_{u}^{\prime }{|}_{\text{u}}\times {\text{F}}_{v}^{\prime }{|}_{\text{u}}}{\left||{\text{F}}_{u}^{\prime }{|}_{\text{u}}\times {\text{F}}_{v}^{\prime }{|}_{\text{u}}|\right|}=s{|}_{\text{u}}\cdot {\text{F}}_{u}^{\prime }{|}_{\text{u}}\times {\text{F}}_{v}^{\prime }{|}_{\text{u}}, \end{array}$$

which is the key information for detailed reconstruction using photometric stereo method.

### 4.3. 2nd-Order Representation

And similarly, the 2nd-order partial derivatives w.r.t. *u* and *v*, respectively are

(11) $$\begin{array}{r} \{\begin{array}{l} {\text{F}}_{uu}^{\prime \prime }{|}_{\text{u}}={\text{T}}_{11}{|}_{\text{u}}\cdot \text{b}\\ {\text{F}}_{vv}^{\prime \prime }{|}_{\text{u}}={\text{T}}_{22}{|}_{\text{u}}\cdot \text{b} \end{array}, \end{array}$$

where **T**_11_|_**u**_ and **T**_22_|_**u**_ denote the matrixes stacking the 2nd-order coefficients w.r.t *u* and *v*, respectively. The 2nd-order information can be used for smooth control during optimization.

Based on face surface embedded with B-spline function, we present the pinpoint 0th- and 1st-order geometric consistency conditions in the following section.

## 5. Consistency Modeling in B-Spline Face Reconstruction

Reconstruction problem is to compute **F** by solving 0th-order consistence of Equation (5) or 1st-order consistence of Equation (6). Generally, two consistency conditions are combined for face reconstruction considering that estimating abundant consistent points in images is limited and that the estimated normals are unfaithful. Furthermore, how to obtain the accurate registration of 0th- and 1st-order information is the most important to high-detailed B-spline reconstruction.

The well-registered textures are low-rank structures of the back projection texture charts. But in practice, they can be easily violated due to the presence of partial occlusions or expressions in the images captured. Since these errors typically affect only a small fraction of all pixels in an chart, they can be modeled as sparse errors whose nonzero entries can have arbitrarily large magnitude.

### 5.1. Modeling Occlusion and Expression Corruptions in 0th-Order Consistence

Let *e*_*i*_ represent the error corresponding to image *I*_*i*_ such that the back projection texture charts $T_{i}={\left(I_{i}◦\tau_{i}^{-1}\right)}^{\#}-e_{i}=T_{i}^{e}-e_{i},i=1,2,\ldots ,n$ are well registered to the surface **F**, and free of any corruptions or expressions. Also combining with 0th-order representation of B-spline face in Equation (7), the formulation (5) can be modified as follows:

(12) $$\begin{array}{r} \begin{array}{l} <\hat{\text{b}},\left\{{\hat{\Pi }}_{i}\right\},\hat{\text{D}},\hat{\text{E}}>=\arg\underset{\text{b},\left\{\Pi_{i}\right\},\text{D},\text{E}}{\text{lim}}||\text{D}|{|}_{*}+\eta ||\text{E}|{|}_{1},\\ s.t.||{\text{D}}^{e}-\text{D}-\text{E}|{|}_{F}\leq \varepsilon . \end{array} \end{array}$$

where ${\text{D}}^{e}=\left[\text{vec}\left(T_{1}^{e}\right),\text{vec}\left(T_{2}^{e}\right),\ldots ,\text{vec}\left(T_{n}^{e}\right)\right]$ and **E** = [vec(*e*_1_), vec(*e*_2_), …, vec(*e*_*n*_)].

However, the solution $\hat{\text{b}}$ of face surface S is not unique if all images are in similar views. And the reconstruction is not high-detailed even if we can make a unique solution by applying a prior face template. So we also need to model high details in 1st-order consistence.

### 5.2. Modeling High Details in 1st-Order Consistence

The resolution of reconstruction is determined by the density of correctly estimated normals. To enhance the resolution of B-spline surface, we use operator (·)^#^ to sample *N*_*p*_ dense parameter points {**u**_*j*_}_*j*=1 : *N*_*p*__ on the domain U for the problem of Equation (6).

Then the well-registered and dense texture are obtained by

(13) $$\begin{array}{r} T_{i}{|}_{{\text{u}}_{j}}={\hat{\text{D}}}_{ji}, \end{array}$$

for *i* = 1, 2, …, *n* and *j* = 1, 2, …, *N*_*p*_.

According to Lambertian illumination model seen in Equation (6), dense normals ***n***_*j*_ as well as light ***l***_*i*_ can be computed from the shading (intensity) of charts $T_{i}$ by SVD method.

Finally, the high detailed reconstruction must satisfy

(14) $$\begin{array}{r} \underset{\text{F}}{\text{min}}\overset{N_{p}}{\underset{j=1}{\sum }}||{\text{n}}_{j}-s{|}_{{\text{u}}_{j}}{\text{F}}_{u}^{\prime }{|}_{{\text{u}}_{j}}\times {\text{F}}_{v}^{\prime }{|}_{{\text{u}}_{j}}|{|}_{2}^{2}. \end{array}$$

By putting Equation (9) into Equation (14), we get

(15) $$\begin{array}{r} \underset{\text{b}}{\text{min}}\overset{N_{p}}{\underset{j=1}{\sum }}||{\text{n}}_{j}-s{|}_{{\text{u}}_{j}}\left({\text{T}}_{1}{|}_{{\text{u}}_{j}}\cdot \text{b}\right)\times \left({\text{T}}_{2}{|}_{{\text{u}}_{j}}\cdot \text{b}\right)|{|}_{2}^{2}. \end{array}$$

Conditions of both Equations (6) and (15) have to be considered for a good reconstruction, which is very difficult. Therefore, we propose a practical solution that combining both 0th- and 1st-order consistence.

## 6. Practical Solution Combining 0th- and 1st-Order Consistence

The problems of both 0th-order consistence and 1st-order consistence are difficult to solve. For , Jacobian matrices w.r.t. ${\left\{\tau_{i}^{-1}\right\}}_{i=1:n}$ have to be computed, which is computing-expensive. And the solution of Equation (15) is not unique, either. Therefore, we aim to find a practical solution to handle both two consistence conditions in this section. We first define the subproblem for each condition, and then provide a iterative algorithm.

### 6.1. 0th-Order Solution

In Equation (6), three kind of parameters including camera parameters {Π_*i*_}_*i*=1 : *n*_, surface parameters **F** (or **b**), and texture parameters ${\left\{T_{i}\right\}}_{i=1:n}$ (or **D**) need to be computed, but they are difficult to be solved simultaneously. We adopt to optimize them by turns, instead.

#### 6.1.1. Estimating Π_*i*_

According to linear transformation from 3D to 2D in Equation (1), we can estimate scale *s*_*i*_, rotation *R*_*i*_ and translation*t*_*i*_ of landmarks for each image *I*_*i*_, *i* = 1, 2, …, *n* based on the and SVD method (Kemelmacher and Seitz, 2011). The image landmarks are detected by a state-of-art detector (Burgos-Artizzu et al., 2013) that has a similar high performance to human. And the 3D landmarks are defined on a B-spline face template with control point parameter **b**_0_, according to Equation (8).

#### 6.1.2. Estimating *b*

Let **f** denote a 2*nf* × 1 vector stacking *f* landmarks of *n* images, and **P** denote a 2*nf* × 3*f* projection matrix stacking *n* views of parameters *s*_*i*_*R*_*i*, [1,2]_, and **t** denote a 2*nf* × 1 vector stacking *f* translation. The update of **b** can be implemented by solving:

(16) $$\begin{array}{r} \underset{b}{\text{min}}||\text{f}-\text{t}-\text{P}\cdot {\text{T}}^{\#l}\text{b}|{|}_{2}^{2}+\zeta ||\left({\text{T}}_{11}^{\#}+{\text{T}}_{22}^{\#}\right)\left(\text{b}-{\text{b}}_{0}\right)|{|}_{2}^{2} \end{array}$$

where the first and the second are 0th- and 2nd-order item, respectively, and ζ is used to balance them. Operator (·)^*#l*^ is a sampling operator that selects B-spline coefficients of landmarks at parameters {**u**(*l*_*i*_)}_*i*=1 : *f*_, and (·)^#^ selects B-spline coefficients at {**u**_*j*_}_*i*=1 : *N*_*p*__. In fact, **T**^*#l*^ is a 3*f* × 3*MN* matrix that stacks **T**|_**u**(*l*_*i*_)_, *i* = 1, 2, …, *f*, and ${\text{T}}_{11}^{\#}$ (or ${\text{T}}_{22}^{\#}$) is a 3*f* × 3*MN* matrix that stacks **T**_11_|_**u**_*j*__ (or **T**_22_|_**u**_*j*__), *j* = 1, 2, …, *N*_*p*_.

The second item also work as a regularization measuring the distance of local information between faces **b** and **b**_0_. It helps eliminate affect of geometric rotation brought by 0st-order warping, and guarantee a smoothness changing during optimization. Particularly, ζ cannot be too small, otherwise a fast changing may bring a local optimal.

#### 6.1.3. Estimating $T_{i}$

$\tau_{i}^{-1}$ and τ_*i*_ is determined by Equation (2) when Π_*i*_ and **b** is known. Then texture chart with noise is obtained by applying consistent parameter sampling $T_{i}^{e}={\left(I_{i}◦\tau_{i}^{-1}\right)}^{\#}$. Let ${\text{D}}^{e}=\left[\text{vec}\left(T_{1}^{e}\right),\text{vec}\left(T_{2}^{e}\right),\ldots ,\text{vec}\left(T_{n}^{e}\right)\right]$. The update of texture charts is to minimize the following formulation

(17) $$\begin{array}{r} \begin{array}{l} <\hat{\text{D}},\hat{\text{E}}>=\arg\underset{\text{D},\text{E}}{\text{lim}}||\text{D}|{|}_{*}+\eta ||\text{E}|{|}_{1},\\ s.t.||{\text{D}}^{e}-\text{D}-\text{E}|{|}_{F}\leq \varepsilon . \end{array} \end{array}$$

which can be solved by Robust PCA (Bhardwaj and Raman, 2016). And let $T_{i}{|}_{{\text{u}}_{j}}={\hat{\text{D}}}_{ji}$, for *i* = 1, 2, …, *n*, and *j* = 1, 2, …, *N*_*p*_.

### 6.2. 1st-Order Solution

Firstly, texture charts based photometric stereo method is used to estimate the local normals. Secondly, a normals driven optimization strategy is proposed to optimize the B-spline face.

#### 6.2.1. Estimating *n*_*j*_

According to Photometric stereo, the shape of each point can be solved by the observed variation in shading of the images. Data of *n* texture charts are input into *M*_*n*×*N*_*p*__ for estimating the initial shape $\tilde{S}$ and lighting $\tilde{L}$ by factorizing *M* = *LS* *via* SVD (Yuille et al., 1999). $\tilde{L}=U\sqrt{\Sigma }$ and $\tilde{S}=\sqrt{\Sigma }V^{T}$, where *M* = *U*Σ*V*^*T*^. To approach the true normal information, we estimate the shape *S* and ambiguity *A* by following the work of Kemelmacher and Seitz (2011). Lastly, the normal at *j*-th point is $n_{j}=S_{j}^{T}$, where *S*_*j*_ is the *j*-th row of *S*.

#### 6.2.2. Estimating b

We normalize ***n***_*j*_ and stack them into a 3*N*_*p*_ × 1 vector **h**. Equation (15) can be rewritten as

$${\text{O}}_{1}=\underset{\text{b}}{\text{min}}||\text{h}-\Lambda {|}_{\text{b}}\cdot (\left({\text{T}}_{1}^{\#}\text{b}\right)⊗\left(\left({\text{T}}_{2}^{\#}\text{b}\right)\right)|{|}_{2}^{2},$$

where Λ is a 3*N*_*p*_ × 3*N*_*p*_ diagonal matrix that stores 3*N*_*p*_ reciprocals of lengths of the normals {***n***_*j*_}_*j*=1 : *N*_*p*__; and (·)^#^ is a selection operator that selects 3*N*_*p*_ rows of 1st-order coefficients at parameter {**u**_*j*_}_*j*=1 : *N*_*p*__; and **b**_0_ represent the control points of a B-spline template face. Particularly, symbol ⊗ denotes a composite operator of cross product, which makes ***w*** ⊗ ***v*** = [***w***_1_ × ***v***_1_; ***w***_2_ × ***v***_2_; …; ***w***_*N*_*p*__ × ***v***_*N*_*p*__], where ***w*** and ***v*** are 3*N*_*p*_ × 1 vectors containing *N*_*p*_ normals.

However, there exists two issues: (1) the low-dimension ***h*** may not guarantee an unique solution of high-dimension ***b***; and (2) the system is not simply linear, which is difficult to be solved. Therefore, a frontal constraint based on template **b**_0_ is applied to make a unique solution; And a strategy of approximating to linearization is also proposed to make a linear solution.

##### 6.2.2.1. Frontal Constraint

The frontal constraint is a distance measurement condition between surface S and template w.r.t. *x*- and *y*-component:

$${\text{O}}_{2}=||{\text{T}}^{\#xy}\left(\text{b}-{\text{b}}_{0}\right)|{|}_{2}^{2}<\epsilon ,$$

where the matrix **T**^*#xy*^ stacks 0th-order coefficients at parameter {**u**_*j*_}_*j*=1 : *N*_*p*__ corresponding to *x*- and *y*- components. Operator (·)^*#sxy*^ also sets the coefficients corresponding to z- components to zeros.

Particularly, the first item ***O***_1_ is not a simple linear form, for which an approximating to linearization is proposed.

##### 6.2.2.2. Approximating to Linearization

According to the characteristics of the cross-product ⊗, the first item in ***O***_1_ can be rewritten as a linear-like formulation:

$$||\text{h}-L{|}_{\text{b}}\cdot \text{b}|{|}_{2}^{2}\text{ or }||\text{h}-R{|}_{\text{b}}\cdot \text{b}|{|}_{2}^{2},$$

where $\{\begin{array}{l} L{|}_{\text{b}}=\Lambda {|}_{\text{b}}\cdot {\left[{\text{T}}_{1}^{\#}\text{b}\right]}_{⊗}\cdot {\text{T}}_{2}^{\#}\\ R{|}_{\text{b}}=-\Lambda {|}_{\text{b}}\cdot {\left[{\text{T}}_{2}^{\#}\text{b}\right]}_{⊗}\cdot {\text{T}}_{1}^{\left(sn\right)}. \end{array}$

Particularly, the operation [·]_⊗_ makes a 3*N*_*p*_ × 1 vector $w={\left[w_{1}^{T},w_{2}^{T},\ldots ,w_{N_{p}}^{T}\right]}^{T}$ become a 3*N*_*p*_ × 3*N*_*p*_ sparse matrix [***w***]_⊗_ = *diag*([***w***_1_]_×_, [***w***_2_]_×_, …, [***w***_*N*_*p*__]_×_), where ${\left[w_{i}\right]}_{\times }=\left[0,-w_{i}^{z},w_{i}^{y};w_{i}^{z},0,-w_{i}^{x};-w_{i}^{y},w_{i}^{x},0\right],i=1,2,\ldots ,N_{p}.$

If **b** is a known parameter, e.g., as **b**_0_, for ***L***|_**b**_, the minimization of ||**h** − ***L***|_***b***_0__ · **b**|| will be a linear system. That is also true for ***R***|_**b**_.

In fact, we can use formulation ||**h** − ***L***|_***b***_0__ · **b**|| to optimize the control points in parameter space of *v* by fixing *u*, and use ||**h** − ***R***|_***b***_0__ · **b**|| to optimize in parameter space of *u* by fixing *v*.

**Algorithm 1:** Iterative Algorithm for B-spline Face Optimization

**Input:** Face images {*I*_*i*_}_*i*=1 : *n*_, B-spline template face **b**_0_, and landmark parameters {**u**(*l*_*i*_)}_*i*=1 : *f*_ in domain U.
1: Detect facial landmark points of images
2: **while b** is not converged **do**
3: **do** // *LOOP1: 0th-order consistence*
4: Estimate camera parameter {Π_*i*_}_*i*=1 : *n*_ according to landmarks.
5: Estimate **b** *via* Equ(16), and update **b**_0_ with **b**. // *Obtain well-registered texture*
6: Register images to texture space by ${\left\{I_{i}◦\tau_{i}^{-1}\right\}}_{i=1:n}$, and build **D**^*e*^ based on unified parameter {**u**_*j*_}_*j*=1 : *N*_*p*__.
7: Solve Equ(17) to obtain $\hat{\text{D}}$.
8: **while** ||**D**||_*_ + η||**E**||_1_ is not converged // *LOOP1 END*
9: Extract texture charts ${\left\{T_{i}\right\}}_{i=1:n}$ from $\hat{\text{D}}$.
10: **while b**_0_ is not converged // *LOOP2: 1st-order consistence* **do**
11: Estimate normals {**n**_*j*_} from $\left\{T_{i}\right\}$.
12: Estimate **b** *via* Equation (18.a), and update **b**_0_ with **b**.
13: Estimate **b** *via* Equation (18.b), and update **b**_0_ with **b**.
14: **end while**
15: **end while**
**Output:** Solution of B-spline objective face **b**.

**Figure 5:**
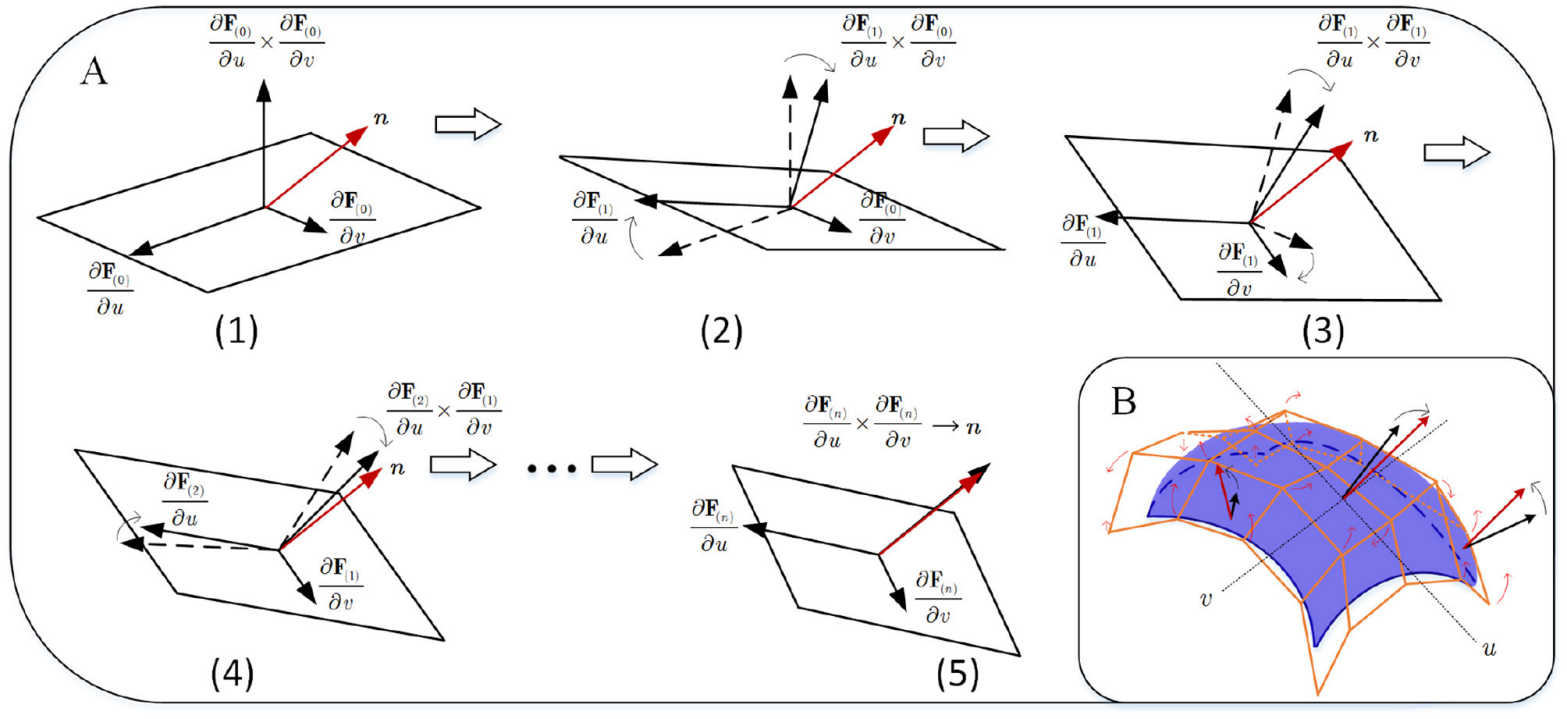
Iterative adjustment on two partial derivatives: Process (1) to (2) adjusts $\frac{\partial F}{\partial u}$ by fixing $\frac{\partial F}{\partial v}$, and process (3) to (4) adjusts $\frac{\partial F}{\partial v}$ by fixing $\frac{\partial F}{\partial u}$, … until that $\frac{\partial F}{\partial u}\times \frac{\partial F}{\partial v}$ is infinitely close to objective ***n***; Process A implements a practically and iteratively linear handle for B-spline surface adjustment in B.

A practical skill is to optimize the control points on *u* and *v* parameter spaces by turns. The two iteration items are rewritten as

$$\{\begin{array}{l} ||\text{h}-L{|}_{{\text{b}}_{0}}\cdot \text{b}|{|}_{2}^{2}+\lambda ||\Lambda_{1}{|}_{{\text{b}}_{0}}\cdot {\text{T}}_{1}^{\#}\cdot \left(\text{b}-{\text{b}}_{0}\right)|{|}_{2}^{2},\\ ||\text{h}-R{|}_{{\text{b}}_{0}}\cdot \text{b}|{|}_{2}^{2}+\lambda ||\Lambda_{2}{|}_{{\text{b}}_{0}}\cdot {\text{T}}_{2}^{\#}\cdot \left(\text{b}-{\text{b}}_{0}\right)|{|}_{2}^{2}. \end{array}$$

where the second term for each formulation is unit tangent vector constraint on the fixed the directions. Λ_1_|_**b**_0__ (or Λ_2_|_**b**_0__) is a 3*N*_*p*_ × 3*N*_*p*_ diagonal matrix that stores 3*N*_*p*_ reciprocals of lengths of tangent vector $\frac{\partial \text{F}}{\partial u}$ (or $\frac{\partial \text{F}}{\partial v}$) at {**u**_*j*_}_*j*=1 : *N*_*p*__. During this procedure **b**_0_ is updated step-by-step. As shown in Figure 5, two partial derivatives $\frac{\partial \text{F}}{\partial v}$ and $\frac{\partial \text{F}}{\partial u}$ at (*u, v*) are updated until $\frac{\partial \text{F}}{\partial v}\times \frac{\partial \text{F}}{\partial u}$ converges to ***n***.

By integrating with **O**_2_, the final formulation of optimization consists of two items as follows:

(18) $$\begin{array}{r} \{\begin{array}{c} \underset{\text{b}}{\text{min}}||\left[\begin{array}{c} \text{h}\\ {\text{T}}^{\#xy}{\text{b}}_{0} \end{array}\right]-\left[\begin{array}{l} L{|}_{{\text{b}}_{0}}\\ {\text{T}}^{\#xy} \end{array}\right]\text{b}|{|}_{2}^{2}+\lambda ||\Lambda_{1}{|}_{{\text{b}}_{0}}\cdot {\text{T}}_{1}^{\#}\left(\text{b}-{\text{b}}_{0}\right)|{|}_{2}^{2},\;\left(a\right)\\ \underset{\text{b}}{\text{min}}||\left[\begin{array}{l} \text{h}\\ {\text{T}}^{\#xy}{\text{b}}_{0} \end{array}\right]-\left[\begin{array}{l} R{|}_{b_{0}}\\ {\text{T}}^{\#xy} \end{array}\right]\text{b}|{|}_{2}^{2}+\lambda ||\Lambda_{2}{|}_{{\text{b}}_{0}}\cdot {\text{T}}_{2}^{\#}\left(\text{b}-{\text{b}}_{0}\right)|{|}_{2}^{2}.\;\left(b\right) \end{array} \end{array}$$

The **b**_0_ is initialized by value of **b**_0_. Then we can solve **b** and update **b**_0_ orderly by minimizing (a) and (b) in Equation (18) iteratively until convergence.

### 6.3. Algorithm

An iterative algorithm is presented for this practical solution in Algorithm 1. Processes of 0th-order consistence and 1st-order consistence are separately conducted in the inner loop. And the outer loop guarantees a global convergence on two consistence problem.

#### 6.3.1. Computational Complexity

The computation in above Algorithm 1 involves linear least square for solving Equations (16), (18.a), and (18.b), SVD for estimating camera parameter, and Robust PCA for Equation (17). In detail, the computational complexity for solving Equation (16) is O(*n*^2^*f*^2^*MN*), and that of both Equations (18.a) and (18.b) are O($N_{p}^{2}MN$). The computational complexity of robust PCA comes to be O($N_{p}^{2}k$), where *k* is the rank constraint. By assuming *N*_*p*_ > *M* > *N* >> *f* > *n*, computational complexity of the other parts can be negligible. In addition, we need considering the number of iteration for total computation of Algorithm 1.

## 7. Experiment

In this section experiments are presented to verify our automatic free-form surface modeling method. We first describe the pipeline to prepare a collection of face images of a person for B-spline face reconstruction. And then we demonstrate the quantitative and qualitative comparisons with recent baseline methods on projected standard images from ground truth 3D data (Zhang et al., 2017) with various expressions, illuminations and poses. Finally, we conduct challenging reconstructions and comparison based on real unconstrained data taken from the challenging Labeled Faces in Wild (LFW) database^1^ (Huang et al., 2007).

### 7.1. Data Pipeline and Evaluation

#### 7.1.1. Synthesized Data With Expression

**Figure 6:**
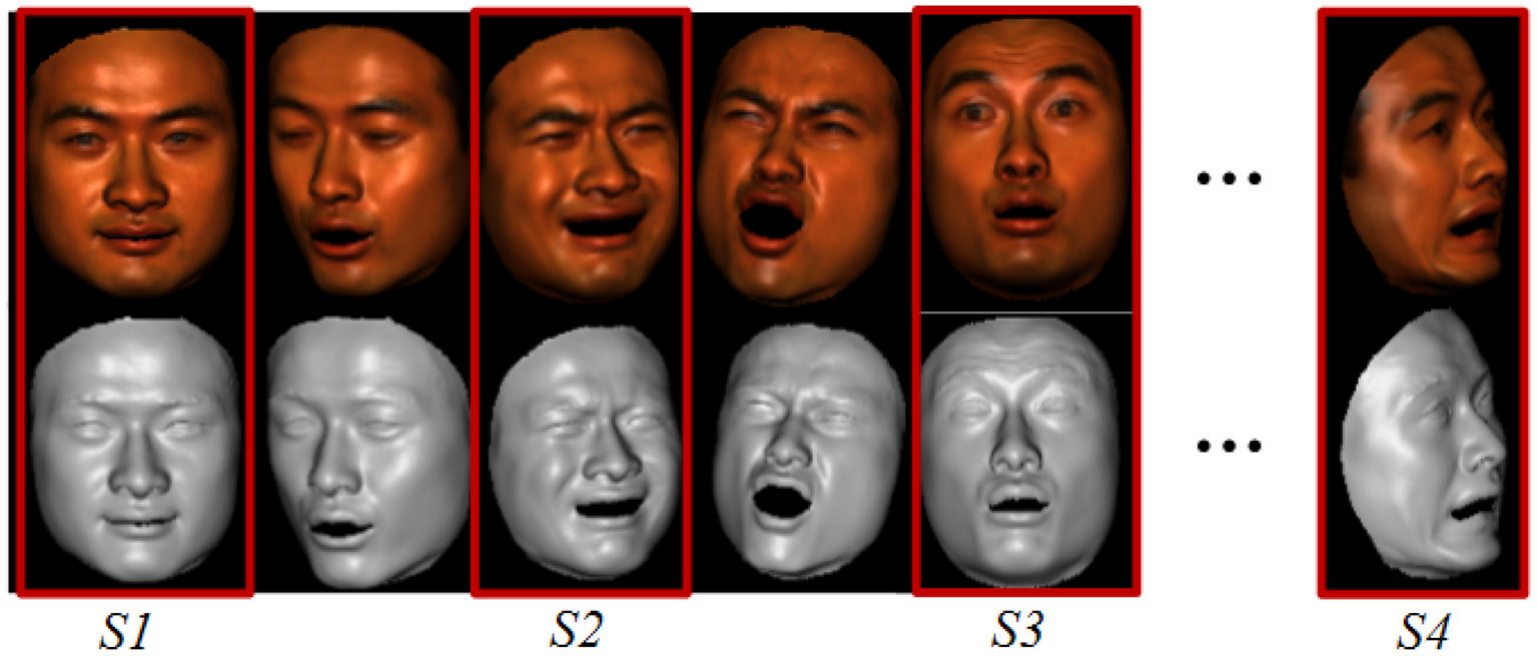
Sample data simulated by the spaces-times faces (Zhang et al., 2017) : images and 3D model with various poses and illuminations are available; data of sample *S1, S2, S3*, and *S4* are used for evaluation.

The ground truth data are from the space-times faces (Zhang et al., 2017) which contains 3D face models with different expressions. We use the data because it is convenient to evaluate our method with ground truth. And different poses and illuminations can also be simulated by the spaces-times faces, seen in Figure 6. Images with various poses and illuminations are collected, and feature points manually labeled. The reconstruction is evaluated by the error to the ground truth model.

#### 7.1.2. Real Data in the Wild

The wild data (Huang et al., 2007) has characteristics of subject variations, illumination changes, various pose, background clutter and occlusions. Images of each person are collected and input into a facial point detector (Burgos-Artizzu et al., 2013) that has a similar high performance to human, to find the 40 facial points shown in Figure 4. The initial B-spline template face is computed from a neutral model of space-time faces.

#### 7.1.3. Comparison

To verify the accuracy of automatic surface reconstruction, discrete points are sampled from the generated continuous free-form shape, and are compared to the traditional discrete reconstructions, e.g., work by Kemelmacher and Seitz (2011) and Roth et al. (2015). For a memory-limited capture system, it is not available to collect thousands of images as what Kemelmacher and Seitz (2011) and Roth et al. (2015) have done, so we limit all the reconstructions to less than forty images. We also compare them with an end-to-end deep learning method by Sela et al. (2017) qualitatively. Deep learning methods rely training on a large amount of unconstrained data, so we just use the model provided by Sela et al. (2017) that have been training on unconstrained images, and test it on the images in the wild.

### 7.2. Synthesized Standard Images

We conduct five sessions of reconstructions: the first four are used to reconstruct expression *S1, S2, S3*, and *S4* by using their corresponding images, and the fifth session *S5* is based on images with different expressions. Each session contains 40 images with various illumination and different poses. Reconstruction results are compared with the re-implemented method Kemel_meth by Kemelmacher and Seitz (2011) and Roth_meth by Roth et al. (2015). Kemel_meth generates frontal face surface based on integration in image domain of size 120 × 110. We clip it according to the peripheral facial points and interpolate points to get more vertices. Roth_meth generates a face mesh based on a template with 23,725 vertices. In our method, control point grid of 102 × 77 is optimized for a B-spline face surface.

#### 7.2.1. Quantitative Comparison

To compare the approaches numerically, we compute the shortest point-to-point distance from ground truth to reconstruction. Point clouds are sampled from B-spline face and aligned according to absolute orientation problem. As done in work of Roth et al. (2015), mean Euclidean distance (MED), and the root mean square (RMS) of the distances, after normalized by the eye-to-eye distance, are reported in Table 1. Particularly, evaluation of Roth_meth is based on surface clipped with same facial points like the other two methods by considering a fair comparison. In the table, the best results are highlighted in boldface, and the underlined result has no significant difference with the best. To our knowledge, Roth_meth is the state-of-art method for face reconstruction from unconstrained images. Its re-implementation version is affected by the noisy normal estimation because of limited number images, showing results that are not good like as in its original paper. But it still performs good on all sessions. As a whole, results by both Roth_meth and our method have lower errors than Kemel_meth. On session S1 and S5, Roth_meth obtains the lowest mean error 5.21 and 6.96%, respectively. However, we obtains lower RMS 4.10 and 4.34% while its errors is quite close to the best especially on session S5. And on session S2, S3, and S4, our method obtains the best results, 6.49 ± 4.66, 4.43 ± 2.91, and 6.46 ± 4.06%. In contrast, the errors by Kemel_meth exceed 8%, and the RMS is also very large on every session. These numerical comparisons supply highly persuasive evidence that our B-spline method can build promising reconstructions based on face images.

**Table 1:** Distances of the reconstruction to the ground truth.

**Meth**.	**Index**	**S1**	**S2**	**S3**	**S4**	**S5**
Kemel_meth	MED (%)	8.08	8.18	8.18	10.75	8.65
	RMS (%)	6.64	6.93	4.29	7.11	6.90
Roth_meth	MED (%)	**5.25**	7.06	5.43	6.63	**6.96**
	RMS (%)	**4.36**	5.79	4.54	4.42	**4.62**
Ours	MED (%)	6.31	**6.49**	**4.43**	**6.46**	6.98
	RMS (%)	4.10	**4.66**	**2.91**	**4.06**	4.34

*The bold means the best value of MED and RMS, while the underline indicates the values next to the best*.

#### 7.2.2. Visual Comparison

**Figure 7:**
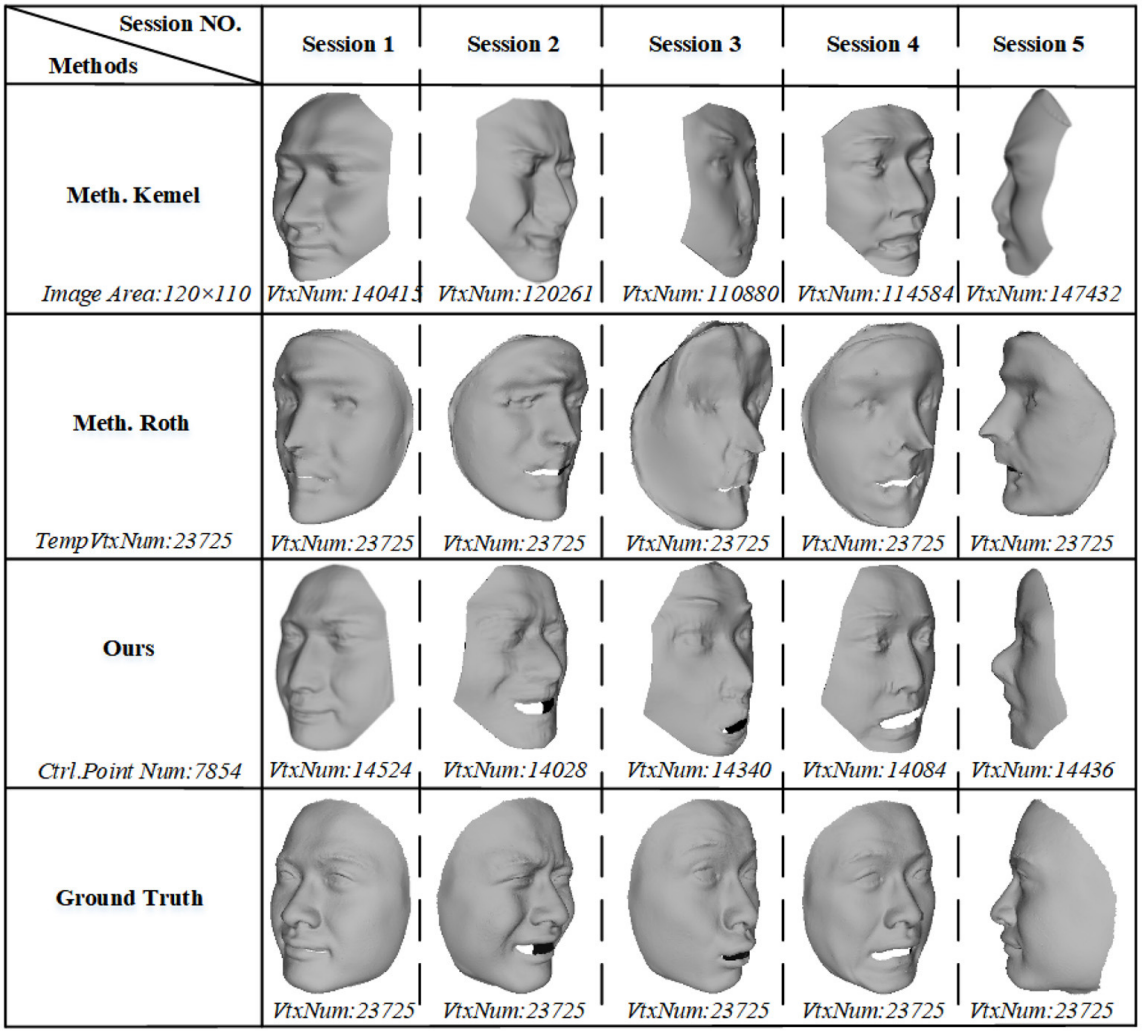
Visual reconstructions and comparisons for session *S1, S2, S3, S4*, and *S5*: for each session of reconstructions, a column lists the 3D results of Kemelmacher and Seitz (2011), Roth et al. (2015), and us, as well as ground truth. (VrxNum means vertex number; TempVtxNum means vertex number of template; and Ctrl.Point Num means the control point number of B-spline face surface. Particularly, the vertices of B-spline face are points sampled from the reconstructed parametric surface).

The visual results in Figure 7. We show 3D models in mesh format for three methods on different sessions, and vertex numbers of models are also presented. It also demonstrates that our method has a promise performance by comparisons in the figure. An important fact is that Kemel (Kemelmacher and Seitz, 2011) cannot make a credible depth information and global shape, e.g., the global shape of reconstruction S2 and the mouse and nose of S3 are obviously incorrect, but our method solves global and local problem by optimization of 0th- and 1st-order consistency. And while Roth (Roth et al., 2015) generates more detailed information of an individual, it also produces distortion at the detailed shape, e.g., the eye of reconstruction S2 and the nose of reconstruction S3 and S4. In contrast, our method obtains realistic shape both globally and locally.

#### 7.2.3. Characteristic Comparison

We give statistics of characteristics of the results generated by the three methods in Table 2, covering the global shape, local detail, credible depth, smoothness, distortion, and derivability. Depending on the quantitative and qualitative comparisons, we also give a rough rating. One star, two stars, and three stars represents bad, general, and good reconstruction respectively in the rating system. Both Roth_meth and our method obtain good scores on global shape, local detail, and credible depth. And both Kemel_meth and our method obtain a good score on smoothness. Because of the bad depth, Kemel_meth also gets bad score on global shape and distortion, and gets general scores on local detail. In addition, B-spline face model has better smoothness than the models by Kemel_meth and Roth_meth, because it is *C*^2^ differentiable parametric surface while the other two are discrete model. Conclusively, 0th- and 1st-order consistency modeling using B-spline surface is efficient to reconstruct parametric surface of individual face.

**Table 2:** A characteristics summarization of three methods by rough rating with number of ✰.

**Characteristics**	**Kemel_meth**	**Roth_meth**	**Ours**
Global shape	✰ × 1	✰ × 3	✰ × 3
Local detail	✰ × 2	✰ × 3	✰ × 3
Credible depth	✰ × 1	✰ × 3	✰ × 3
Smoothness	✰ × 3	✰ × 2	✰ × 3
No distortion	✰ × 1	✰ × 2	✰ × 3
*C*^2^ differentiable	NO	NO	YES

### 7.3. Real Unconstrained Images

Our method is also tested based on real unconstrained data. Unconstrained data mean that the images are captured under uncertain condition, and the faces in the images are different in expression, pose and illumination condition. It is difficult to build the geometrical consistency for reconstruction using such data. Unlike the experiments in the work by Kemelmacher and Seitz (2011) using hundreds of images, we conduct reconstruction with limited number of images, because a large mount of face images for one person are not always available for small sample size tasks such as criminal investigation. In the experiment, uniformly 35 images are collected for each person from LFW database^1^ covering different poses, illuminations and expressions.

**Figure 8:**
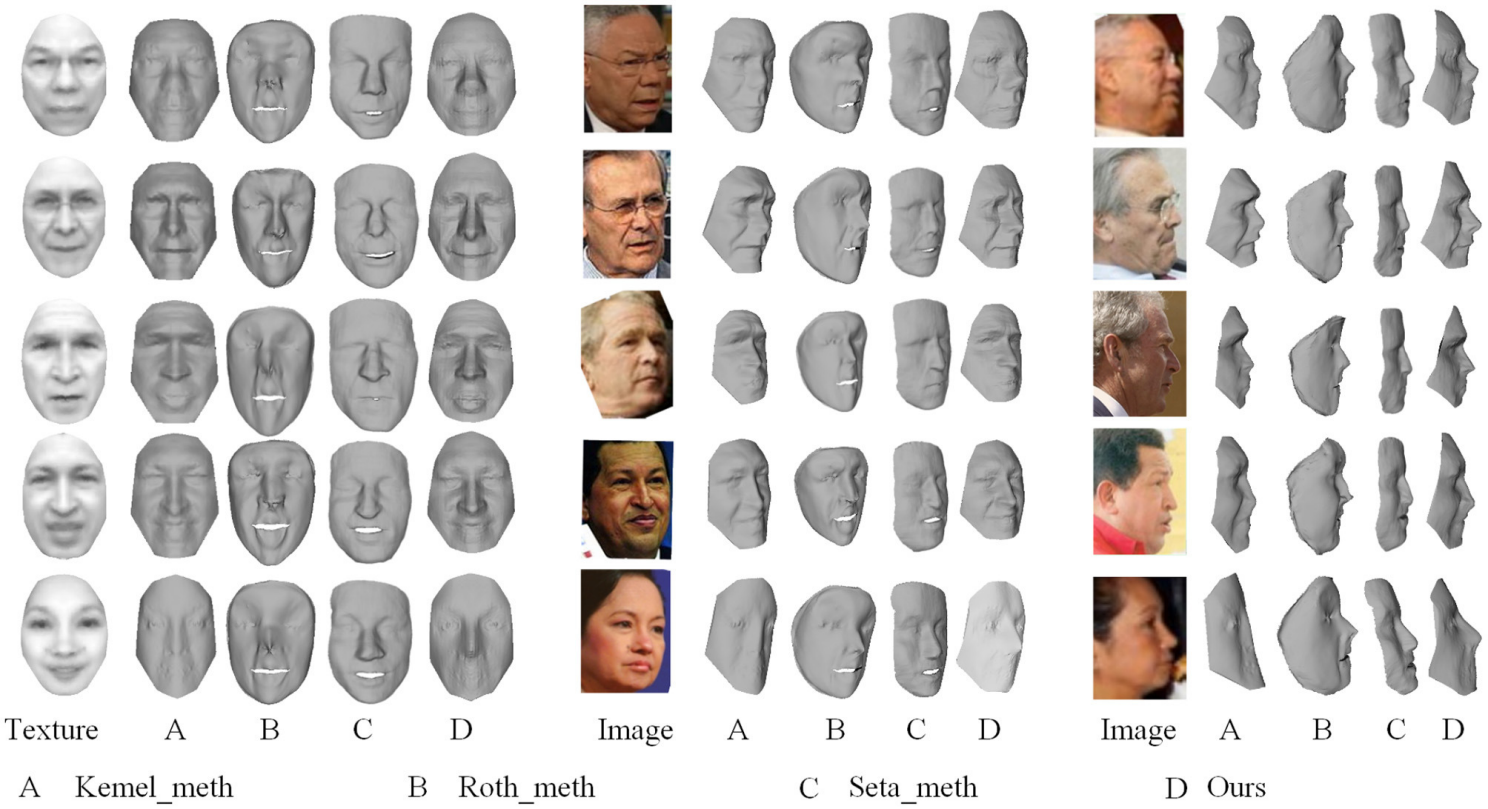
Visual reconstructions and comparisons for Colin Powell, Donald Rumsfeld, George W. Bush, Hugo Chavez, and Gloria Macapagal Arroyo: (1) Images in column 1, 5, and 9 are corresponding mean textures and two views of images, respectively; (2) Columns labeled by *A* show the results generated by the reimplemented Kemel_meth, and columns labeled by *B* show the results generated by the reimplemented method Roth_meth, and columns labeled by *C* show the results generated by the Seta_meth, and columns labeled by *C* show our results. (The face images used in the figure come from LFW database^1^).

Visual face reconstructions for Colin Powell, Donald Rumsfeld, George W. Bush, Hugo Chavez, and Gloria Macapagal Arroyo are compared with other two methods, as shown in Figure 8. Let *A* label the results generated by the reimplemented Kemel_meth, and let *B* label the results generated by the reimplemented Roth_meth, and let *C* label the method Seta_meth of deep learning by Sela et al. (2017) and let *D* label our results. Particularly, the input for Seta_meth is one image selected from the 35 images. Images in column 1, 5, and 8 are corresponding mean textures and two views of images respectively. By comparing these results, we observe some phenomena as follows:

1. In frontal viewpoint, *A* and *D* show more vivid details than *B*, e.g., eyes and nose of Colin Powell. But in an other viewpoint, *D* shows more credible shape than *A*, e.g., the eyes and the forehead of Colin Powell, and the forehead and the mouth of Donald Rumsfeld.
2. When the normals are incorrectly estimated from a limited number of images, e.g., for Gloria Macapagal Arroyo, *A* loses the local information completely, but *B*, *C*, and *D* still maintain general geometrical shape of face. For all methods, reconstructing nose is a challenge because the geometric curvature of the nose varies greatly. When the images are not enough, the noise could be amplified. So *B* shows bad results at nose being limited by number of input images.
3. The input of *C* is a approximately frontal face image selected. As the model of *C* is learning on a set of 3D face data, it may not handle the uncertain noise and identity of inputs. So the details in reconstruction by *C* don't look real, although their global shapes are stable and like human faces.
4. By comparison, our method steadily produces better looking results than others from different viewpoints in the dataset. Clear and vivid details can be seen at key components such as eyes, nose and mouth, forehead, and cheek.

## 8. Discussion

All the above experiments prove that our method can build pinpoint geometrical consistency on the limited number of real unconstrained data. Our method may not be best method in area of 3D reconstruction from multiple images, as the results in the original work by *B* looks better. It could deal with 3D reconstruction with limited number of images. Because we may not obtain large amount of images for reconstruction as done by Roth et al. (2015), for some condition restricted system. The shortcomings of *A* are mainly resulted from the inauthentic depth generated by integration method. And the bad results of *B* are caused by that the mesh template cannot build correct geometric consistency of number limited of unconstrained images and that the discrete differential operating on estimated noisy normal brings distortion errors. In contrast, we build pinpoint geometric consistency using B-spline surface. B-spline can smooth the noise in estimated normal better. So *D* can reconstruct correct face shape with little distortion, showing better result as a whole.

In the comparison, we don't consider other deep learning methods based methods appeared in recent years (Dou et al., 2017; Richardson et al., 2017; Lin et al., 2020; Sengupta et al., 2020; Shang et al., 2020). Because almost all recent works are focused on deep learning methods for single image based 3D face reconstruction (Dou et al., 2017; Richardson et al., 2017; Lin et al., 2020; Sengupta et al., 2020), as well as using a 3DMM model as prior. And the multi-view deep learning method only handle constrained face images (Shang et al., 2020). It means the deep learning methods can use a large amount of training data, and also a good prior. The input are different between these learning based methods and our method. So we conduct comparison with the classic optimization-based approaches for the sake of fairness. Nevertheless, we also select one representative method by Sela et al. (2017) to show result by deep learning as a reference in the comparison. It proves that if the test are not satisfactory to the prior and distribution of training data, it may obtain bad result.

## 9. Conclusions

This study set out to present high-detailed face reconstruction from multiple images based on pinpoint 0th- and 1st-order geometric consistence using B-spline embedding. Based on the good consistence modeling in geometric optics, the method works well for data with different poses and expressions in the wild. The key contribution of this study is that surface modeling adapts the correct rays in geometric optics by using B-spline embedding. This makes the high-detailed B-spline modeling from a number limited of face images captured under wild condition become reality. The method could also be applied to expression tracking and assisting face recognition in a monitoring or robot system.

## Data Availability Statement

The original contributions presented in the study are included in the article/supplementary material, further inquiries can be directed to the corresponding author/s.

## Author Contributions

WP and ZF has contributed equally to the core idea as well as the experiment design and results analysis. YS, KT, and CX has provided assistance in experiments and analysis, under ZF's supervision. Besides, KT and MF provided the research group with financial support and experimental equipments. KT and ZF are supportive corresponding authors. All authors contributed to the article and approved the submitted version.

## Conflict of Interest

The authors declare that the research was conducted in the absence of any commercial or financial relationships that could be construed as a potential conflict of interest.

## Footnotes

^1^http://vis-www.cs.umass.edu/lfw/
